# Impact of Molar Teeth Distalization by Clear Aligners on the Temporomandibular Joint: Systematic Review and Meta-Analysis

**DOI:** 10.3390/jcm14165836

**Published:** 2025-08-18

**Authors:** Kacper Galant, Sylwia Dąbrowska, Natalia Turosz, Konrad Małkiewicz

**Affiliations:** 1 Student Scientific Club of the Department of Orthodontics, Medical University of Lodz, 251, Pomorska St., 92-213 Lodz, Poland; kacpergalant.ld@gmail.com (K.G.); sylwiadabrowska199@gmail.com (S.D.); 2 National Medical Institute of the Ministry of Interior and Administration, Wołoska 137 Str., 02-507 Warsaw, Poland; natalia.turosz@gmail.com; 3 Department of Orthodontics, Medical University of Lodz, 251, Pomorska St., 92-213 Lodz, Poland

**Keywords:** clear aligners, molar distalization, temporomandibular joint

## Abstract

**Background:** This review aimed to assess the association between upper molar distalization using clear aligners (CAs) and structural changes in the temporomandibular joint (TMJ). **Methods:** On February 20, 2025, the following databases were searched: PubMed, Embase, BASE, Web of Science, and Scopus. Studies were included if they reported on orthodontic treatment with CAs involving upper molar distalization and presented TMJ parameter measurements before and after treatment. The JBI Critical Appraisal Checklist for Quasi-Experimental Studies was used to assess the risk of bias. The collected data were analyzed using the paired t-test, mean difference assessment, and Spearman correlation matrix. **Results:** A total of 4 articles out of 238 records retrieved were included in the review. The mean age of patients was 23.18–29.80 years, and the treatment duration was 1.90–2.21 years. The most important changes were in the posterior and superior joint spaces (PJS and SJS) (*p* < 0.05), with SJS increasing in two studies (0.56 and 0.7 mm) and PJS increasing in one and decreasing in another (−0.94 and 0.36 mm). **Conclusions:** Limited evidence suggests that molar distalization with CAs may influence TMJ dimensions to a small extent. However, the results are inconsistent and require further validation with high-quality studies to draw firm conclusions. **Registration:** The review was pre-registered using the OpenScience Framework (OSF) on 17 April 2025—osf.io/9xyr8. No funding or conflicts of interest were reported.

## 1. Introduction

### 1.1. Rationale

Class II malocclusion is a common orthodontic defect worldwide [1,2]. There are many available methods for treating this condition; one of them is upper molar distalization [3]. Molar distalization is often undertaken to create 2–3 mm of space in the dental arch, gaining a Class I dento-alveolar relationship [4,5]. Correction of such a defect in adult patients with this treatment method is primarily due to tooth movement without or with minor secondary changes in soft tissue and skeletal components [6,7]. This can be achieved using orthodontic appliances called clear aligners (CAs).

The possibility of using clear thermoplastic overlays in orthodontic treatment has evolved since its initial concept by Harold Dean Kesling in 1945 and officially presented in 1946 [8]. They gained a significant boost in popularity when Align Technology introduced the first commercial system, Invisalign, in 1999 [9]. FDA approval of this system in 2000 further increased its popularity [10].

CAs are made from transparent, flexible plastic materials, and they apply controlled light forces to move teeth into predetermined positions [11]. Each aligner should be worn at least 22 h a day to obtain satisfactory and intended results [12]. According to the attending physician, aligners are usually replaced every 1–2 weeks [13].

Currently, orthodontic treatment using CAs is increasingly chosen by patients, mainly because they are more aesthetic and comfortable than conventional fixed appliances (FA) [14,15].

Accelerated progress in the technology which is used to manufacture these appliances results in greater possibilities in the treatment of more orthodontic defects and more complex malocclusions [16]. CAs are a well-regarded option for addressing dental issues like crowding, spacing, overbites, underbites, and open bites [9]. However, it is important to remember that there are certain limitations to the severity of defects. The most suitable will be minor to moderate defects [17,18,19]. The continuous development of 3D printing and digital scanning technology gives hope for the dynamic development of CAs in the future, allowing for increasingly precise and accurate treatment plans [20,21].

Aligners cover entire tooth crowns, which facilitates tooth movement and affects the opening of the occlusion and the release of the mandible [5,22].

Modern treatment of Class II malocclusion using CAs requires a biomechanical strategy such as attachments, elastics, and thorough, careful planning of tooth movement. Only in that way can we achieve predictable motion, simultaneously preventing side effects [5,22,23]. Properly designed attachments and elastics allow for avoidance of tipping, loss of anchorage, and potential flaring of the anterior teeth [5].

Sequential movement planning is also essential [5,22,23]. It consists of arch distalization with staging set at 0.25 mm per aligner [5,23]. Precise movement control and a staged approach minimize the risk of complications such as mandibular rotation [23]. CAs enable to achievement of precise tooth movement—crown and root displacement of approximately 2.23 mm [5]. Moreover, proper planning and the use of correct protocols allow for almost 90% accuracy in molar distalization [11,24].

The use of traditional devices within this type of movement, such as distal jets or a pendulum appliance, due to the application of force at a distance from the center of resistance, creates a moment that results in distal tipping and extrusion of molars. Applying force also causes loss of anchorage [25,26,27]. In CA treatment, the use of appropriately shaped attachments—rectangular and vertical on the buccal surface of the teeth—and the additional use of Class II elastics helps reduce tipping and uncontrolled proclination of the anterior teeth [28,29].

Even though there are many articles in the scientific literature regarding the advantages and effectiveness of CAs, relatively few of them focus on their impact on the temporomandibular joint (TMJ) [22]. The relationship between orthodontic treatment of malocclusions and temporomandibular disorders (TMDs) is clinically significant [22,30]. Additionally, further research is needed to investigate the long-term effects of CA use [22].

TMDs can have many clinical manifestations, including pain, joint sounds such as popping or clicking, and restricted movement of the temporomandibular joints. It can also affect adjacent structures [30,31,32]. These disorders most often occur in the second decade of life [31], particularly among females [30]. Orthodontic treatment can directly affect the structures in the joint, which can initiate or exacerbate symptoms or impair function [30,31,32]. Therefore, researchers emphasize the importance of thorough assessment for any signs of TMD before starting treatment. Additionally, prolonged treatment may lead to more advanced changes [31].

Using CBCT scans before and after treatment enables accurate assessment of morphological parameters and may improve our understanding of TMJ pathology [33,34,35,36].

Due to the increased popularity of orthodontic treatment [14,15], analyzing the problem we have chosen is crucial.

### 1.2. Objectives

This study aims to evaluate the impact of distalization of molar teeth by clear aligners on the temporomandibular joint, based on available evidence from published research.

## 2. Materials and Methods

### 2.1. Eligibility Criteria

This systematic review included data from patients who underwent orthodontic treatment with aligners. The patients had their molars distalized, and joint measurements were taken before and after treatment using imaging studies such as CBCT or MRI. More detailed eligibility criteria are presented in Table 1.

Distalization was performed on both Class I and Class II patients. However, in Class II cases, it was the only method used to correct the molar relationship, without any additional interventions such as extractions or functional appliances.

### 2.2. Search Strategy

The review was based on a query created by one of the authors:

(aligner OR aligners) AND (orthodontic OR clear OR invisible OR invisalign OR transparent) AND (distalization OR retraction) AND molar AND (TMJ OR temporomandibular OR joint OR condyle OR glenoid OR fossa OR coronoid OR condylar OR process). The other authors approved it.

### 2.3. Information Sources

It was used to search the following databases: PubMed, Embase, Scopus, Web of Science, and Bielefeld Academic Search Engine (BASE), which was performed on 20 February 2025 (Table A1 in Appendix A). The query is based on synonyms defining orthodontic aligners, required tooth movement, and anatomical structures, whose changes were sought.

The authors also attempted to manually search for articles using popular search engines, which resulted in positive results in the form of two further publications (Al-Somairi et al. 2025 [23]; Zheng et al. 2023 [22]).

### 2.4. Selection Process

Manual deduplication was performed. Then, the authors executed initial screening of the titles and abstracts to identify appropriate publications using blinded screening in the Rayyan tool (version 2024-04-18; Qatar Computing Research Institute, Doha, Qatar, and Rayyan Systems, Cambridge, MA, USA). The publications were reviewed and qualified or disqualified based on the previously mentioned eligibility criteria. Subsequently, the authors evaluated the full text of the chosen articles. This systematic review includes citations exclusively from carefully selected studies. Rejected papers were noted in this article with the reason for exclusion (Table A2 in Appendix A). MedCalc program (Version 23.2.1; accessed 12 April 2025) was used to calculate the Cohen’s κ coefficients to assess the agreement between evaluators. The inter-rater agreement was κ = 0.66, corresponding to moderate to good compliance.

### 2.5. Data Collection Process

Manual data extraction and export of the obtained numerical data into a spreadsheet using Google Workspace software (Version 2024.08.23; Google LLC, Mountain View, CA, USA) were performed.

### 2.6. Data Items

The following parameters were collected: (1) number of patients, (2) age of participants, (3) type of imaging used, (4) mean time of the treatment, (5) class of malocclusion before treatment, (6) MFH—Mandibular Fossa Height, (7) MFW—Mandibular Fossa Width, (8) CL—Condylar Length, (9) CW—Condylar Width, (10) AJS—Anterior Joint Space, (11) SJS—Superior Joint Space, (12) PJS—Posterior Joint Space, and (13) MJS—Medial Joint Space. Specific joint parameters and methods of their measurement, as well as differences in these measurements between authors, are presented in Table 2.

### 2.7. Study Risk of Bias Assessment

The authors assessed methodological behavior in eligible articles using the Joanna Briggs Institute (JBI) Critical Appraisal Checklist for Quasi-Experimental Studies [37]. This tool comprises nine criteria, including assessment of temporal precedence, selection and allocation of participants, confounding factors, administration of the intervention/exposure, outcome measurement, participant retention, and statistical validity. To increase the transparency of the assessment, a traffic light plot and summary plot were created illustrating the judgments for each included study across all domains using the Robvis tool [38].

### 2.8. Effect Measures

MedCalc Software Ltd. Comparison of means calculator (Version 23.2.1; accessed 11 April 2025) was used to analyze the obtained results, which were used to calculate mean differences, standard error, and 95% confidence interval and significance level. Correlation coefficients were included as additional effect measures.

### 2.9. Synthesis Methods

The evaluation of the correlation between mean differences for different parameters was performed by creating a correlation matrix based on the Spearman correlation test. The Shapiro–Wilk test was used to analyze the normality of the data distribution, and then the Student’s *t*-test for dependent samples was performed. Statistica software (version 13, TIBCO Software Inc., Palo Alto, CA, USA) was used for statistical analysis. The significance level adopted was *p* = 0.05.

### 2.10. Registration

The study was preregistered using OSF registries (osf.io/9xyr8) on 17 April 2025.

## 3. Results

### 3.1. Study Selection

The initial search included 239 records: 137 from PubMed, 45 from BASE, 18 from Scopus, 18 from Web of Science, and 18 from Embase, and the other 2 were located through manual searching. A total of 83 articles were removed before screening due to duplication. The authors screened 156 articles, of which 4 were jointly qualified.

**Figure 1:**
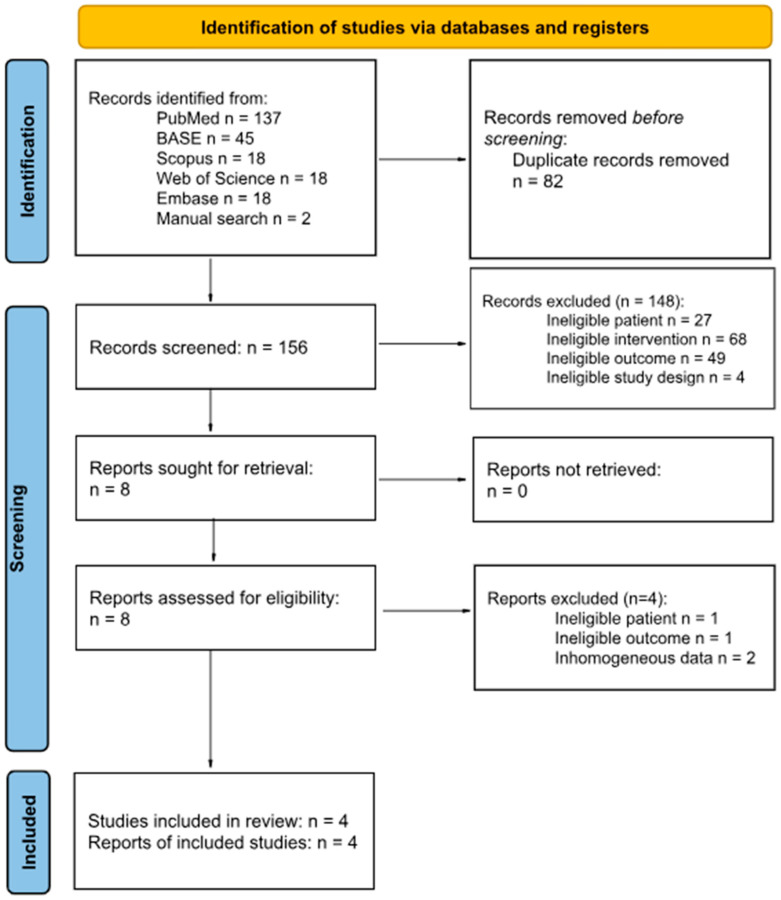
Flow diagram.

Details of this process are presented in Figure 1. Records rejected at the full-text stage are listed with the rejection reason in Table A2.

### 3.2. Risk of Bias in Studies

**Figure 2:**
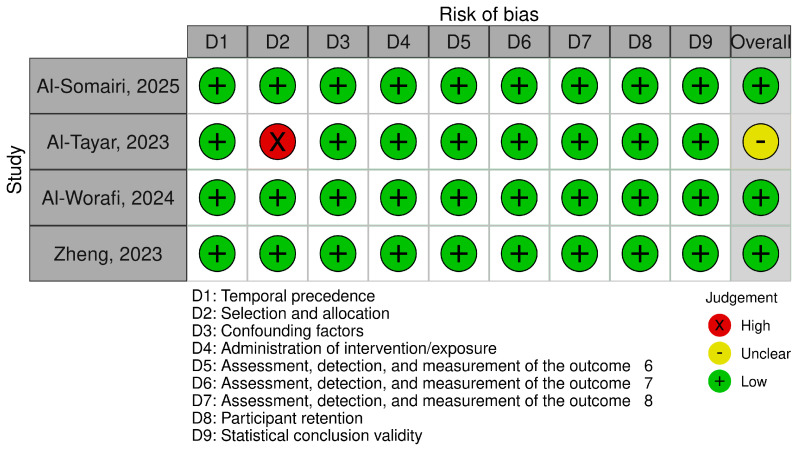
A graphical representation of evaluations at the domain level for each individual study [5,22,23,36].

**Figure 3:**
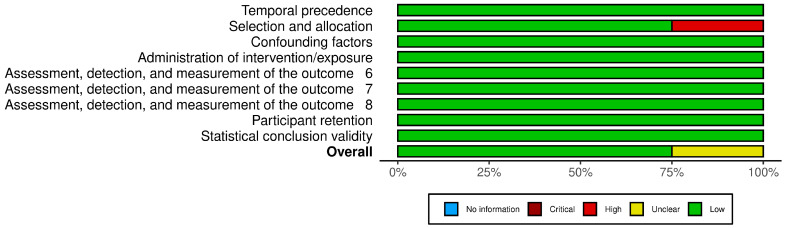
Bar visualizations representing the distribution of risk-of-bias evaluations across each domain.

Of the four included articles, three were assessed as low risk of bias, with all nine criteria fully met. One of them was assessed as moderate risk due to concerns related to participant selection and allocation (Table 3). Figure 2 and Figure 3 summarize RoB assessments using the traffic-light plot and summary plot.

### 3.3. Results of Individual Studies

The review ultimately included four studies (Table 4). The mean age of the study participants was 25.73 years. The treatment time was 2.03 years, and the studies included mainly patients with Class II malocclusion. The remaining results regarding the dimensions of selected joint parameters are included in Table 5 and Table 6.

### 3.4. Results of Syntheses

To evaluate the obtained results presented in Table 5 and Table 6, the assessment started with analyzing the normal distribution of these data using the Shapiro–Wilk test, which indicated that all the data defining the dimensions of the joint and its surroundings were normally distributed. Then, the *t*-test for dependent samples was used to evaluate these parameters, which revealed no statistically significant relations before and after the treatment, irrespective of the selected parameter.

Then, the means of individual parameters before and after treatment were compared individually for each parameter and author. The results are presented in Table 7.

Al-Tayar et al. and Zheng et al. reported statistically significant differences in the dimensions defining the Posterior Joint Space [22,36]. Al-Tayar et al. [36] obtained the greatest reduction of this space value (Md = −0.94), while Zheng et al. [22] obtained the greatest enlargement of this space (Md = 0.36), suggesting posterior displacement or anterior displacement of the mandibular head, respectively.

The same two authors also revealed significance (*p* < 0.0001) for the superior joint space. Both obtained an increase in this space, greater for Zheng et al. (0.7 vs. 0.56) [22], which could suggest a downward displacement of the mandible. In the case of the Anterior Joint Space, only Zheng et al. [22] obtained a statistically significant result. The negative result obtained by this research team (Md = −0.22) indicates posterior displacement of the condyle.

In the scope of the mandibular fossa (MFW) dimensions, statistically significant differences were reported only in the study by Al-Tayar et al. (*p* < 0.0001) [36], demonstrating an increase in the mean value (Md = 2.61), which may indicate bone remodeling in the area of the joint socket. The same author also obtained the only statistically significant result (*p* < 0.0001) for changes in the dimensions of the articular condyle (Md = −0.74 mm), implying resorptive changes.

In the case of MFH, CL, and MJS, no authors reported statistically significant results (*p* < 0.05).

The Spearman correlation test was used to assess the correlation between changes in the values of individual variables (Table 8). This enabled identification of several strong correlations: PJS—MFH; PJS—CW; MFH—CW; and SJS—CL (ρ = −1.0), where an increase in one variable corresponded with an increase in the other, and inverse correlations are as follows: PJS—AJS; AJS—MFH; and AJS—CW (ρ = −1.0), where an increase in one value was associated with a decrease in the other. Unfortunately, all obtained correlation coefficients (even those indicating strong relationships) failed to reach statistical significance (*p* > 0.05).

### 3.5. Reporting Biases

Given the limited number of studies (*n* = 4), a formal assessment of reporting bias, such as funnel plots or statistical tests for asymmetry, was not possible to perform. Therefore, the risk of bias due to missing results could not be reliably assessed.

## 4. Discussion

In the obtained results, special attention should be paid to the changes occurring in PJS and SJS because these are the only statistically significant changes obtained by two authors simultaneously. While Al-Tayar et al. [36] observed an increase in the PJS, Zheng et al. [22] reported a decrease in this dimension, highlighting inconsistent outcomes between studies. In contrast, in the case of SJS, both teams noted an increase in the dimensions of this space, with results showing high statistical significance (*p* < 0.0001).

Changes in the contact points between the opposing dental arches appear to be associated with adaptation at the neuromuscular level, with altered muscular activity, which is temporary and reversible, and alterations in mandibular positioning relative to the maxilla instead of reflecting bone remodeling, resorption, or repositioning [39,40]. To optimize occlusal contacts, the neuromuscular system can shift the position of the mandibular condyle downward, which corresponds to an increase in SJS [41]. Only a limited number of studies reported statistically significant changes in the dimensions of the mandibular fossa or the condylar process (*p* < 0.05). This could be attributed to the age of the study population, with a mean age above 23 years, the stage during which craniofacial growth is minimal or complete. This is also due to the device’s purpose, which is not to enhance or redirect the growth of the mandible to correct the skeletal discrepancy but to camouflage the underlying discrepancy and achieve improvement at the alveolar level.

It can also be speculated that the increase in occlusal space caused by a certain aligner thickness causes mandibular displacement and an increase in SJS. This is called the occlusal splint effect of the clear aligner [42].

Studies that assessed changes in the joint in younger patients with a certain growth potential and used functional devices aimed at modifying the patient’s growth showed statistically significant changes not only in the joint spaces but also remodeling in the condylar process and glenoid fossa [43,44].

In the case of patients with a Class II malocclusion, there are several methods of treatment depending on the patient’s financial capabilities, the patient’s profile, and the severity of the defect. We can (1) accept the defect, (2) perform extraction treatment, (3) perform non-extraction treatment using distalization, or (4) perform orthognathic surgery in the case of significant, severe defects [45,46,47].

The results are consistent with those obtained by Samra et al. [48], where the distal-jet device was used primarily for molar distalization. The authors analyzed changes in the TMJ in a group of patients of similar age and also obtained statistically significant changes in the SJS and PJS, but in the case of both of these spaces, an increase in values was observed.

In the case of extraction treatment of Class II malocclusions, Alhammadi et al. [49] obtained statistically significant changes for AJS and PJS, where the former increased in size and the latter decreased.

As indicated by Vogl et al. [50], in the case of surgical treatment of Class II defects, statistically significant changes also occur in the upper and Posterior Joint Space, but the results obtained are opposite to those for distalization using CAs; there is a reduction in dimensions of these spaces. This author also obtained a statistically significant reduction in MJS and LJS.

Patients treated by the authors who showed statistically significant changes in the PJS and SJS dimensions were Class II patients. Particularly in Class II subtype II, the mandible is pushed posteriorly, causing it to assume a posterior position. Only incisor protrusion and correction of deep overbite allow the mandible to “unlock” and move forward and change position, which would correspond to increased PJS and SJS, according to our results [22,36,49].

Distalization of the upper molars is associated with side effects in the form of clockwise rotation of the mandibular plane and a decrease in the ratio of posterior to anterior facial height [25,51]. This is why it is necessary to properly assess and correct the position of the upper first molars in the sagittal plane to maintain optimal vertical dimension and functional occlusion [52]. This constitutes a contraindication to the treatment of hyperdivergent patients for fear of further deterioration of the lateral profile. Such changes in the cephalometric analysis would correspond to our results in the form of an increase in SJS and a probable decrease in PJS. However, studies indicate that CA treatment does not significantly affect changes in parameters such as the SN-MP angle and anterior and posterior facial height [53,54], allowing for better control of mandibular divergence during molar distalization. This is also reflected in the results, as the statistically significant changes in SJS and PJS appear too minor to produce any clinically perceptible effect on the patient’s facial profile.

Mandibular posterior rotation in distalization treatment results from distal tipping of the molars and downward displacement of the mesial cusp, resulting in the previously mentioned profile changes [55,56,57].

The use of staging and the introduction of attachments during distalization, which create a counter-moment that counteracts tipping, allows for achieving almost 90% movement accuracy [11,24]. This may be the reason for the lack of significant changes in TMJ dimensions in the presented results. However, errors in aligner fabrication, incorrect attachment placement, and improper treatment planning can promote undesirable movements and cause some changes in the TMJ. Elastics, which are often used, also promote molar extrusion, contributing to undesirable mandibular rotation. Extrusion is also counteracted by the thickness of the aligner and the use of aligner chews, causing a bite block effect and enhancing the intrusive effect [58]. Correct treatment planning and force configuration are key to the success of overlay treatment and the absence of changes in the vertical dimension [58]. It is necessary to point out here that the potentially occurring changes in the dimensions of the TMJ in such a case are not the result of the direct action and influence of the CA but their indirect influence through changes in occlusal contacts.

Class II malocclusion is less frequently associated with TMD than Class I, but the symptoms are more severe. These patients are more likely to have arthralgia, myalgia, and restricted mouth opening compared to those with Class I [59]. It has been suggested that features associated with Class II patients, such as retrognathic mandible and hyperdivergent growth pattern, are also associated with TMJ disc displacement [60].

As indicated by Yu et al., increased AJS and decreased SJS and PJS, i.e., results opposite to those obtained in this review, occur mainly in patients with disc displacement [61]. This suggests a potential benefit for patients with such a problem, treated with CAs in the form of distalization, eliminating TMD.

It should also be taken into account that CA treatment may be a factor responsible for the development of TMJ symptoms, especially in patients who poorly adapt to occlusal changes [62]. Unfortunately, there is still no clear answer as to whether FAs or CAs would be a better treatment for patients with joint disorders due to mixed opinions in research articles [63].

Distalization of molars using CAs may also affect other areas of our body, such as the upper respiratory tract, and lead to disturbances in fluid dynamics, potentially raising susceptibility to pharyngeal collapse [64].

The results obtained in the Spearman correlation matrix suggest a potential relationship between individual parameters; unfortunately, due to the lack of statistical significance (*p* > 0.05), they are only exploratory, which may be due to the limited number of studies and differences in research methodology. However, they indicate a certain trend and warrant further investigation on larger samples to confirm their clinical significance.

The small number of cases and the data presented in the articles directly affect the conducted analysis and its low statistical power. Some studies showed variability of selected measurement points to determine the dimensions for the desired parameters, which disturbs the homogeneity of the obtained results and introduces errors within the measurement methods and the quality of the obtained data. The lack of homogeneity in patient selection and the use of distalization in Class I patients (not only Class II) to correct dental irregularities, such as crowded teeth, also negatively impacts the validity of the results. Further studies are necessary, including long-term follow-up assessments with the use of a control group to compare outcomes, which would provide long-term outcomes of such treatment and further progressive adaptive mechanisms.

The results in the correlation matrix may indicate strong relationships between selected parameters. Still, the current lack of significance requires further verification in studies with a larger group of participants and a longer observation time.

Studies on this topic require development towards a larger number of prospective studies allowing for more detailed monitoring during treatment and the use of other imaging techniques, e.g., MRI, which does not expose the patient to additional risk related to radiation and perfectly visualizes soft tissues—the joint disc, also a very important structure.

The findings of this review may have clinical importance when selecting a treatment method for patients with Class II malocclusion. Distalization of upper molars using CAs possibly affects functional changes, not structural changes in the TMJ, which emphasizes the relative safety of the method used in adult patients with completed growth. Changes in the position of the mandibular condyle may not manifest clinically. In patients without TMD, this method may be preferred over FAs due to the control of the applied force, resulting in minimization of sudden alterations in occlusion and gradual neuromuscular adaptation. In turn, in patients with TMJ dysfunction, the observed increase in space—especially the upper and Posterior Joint Space—may reduce the load and pressure on the condyle, alleviating the disc-related symptoms. However, success in therapy—regardless of the method—relies on individualized protocols, accurate patient selection, as well as comprehensive TMJ assessment, both clinical and imaging, as well as monitoring of the temporomandibular joint during and after treatment at follow-up visits.

## 5. Conclusions

The limited available results presented in this review indicate minor changes in TMJ structures, mainly in the SJS and PJS. These changes appear to be consistent with neuromuscular adaptation rather than skeletal remodeling. Further studies with standardized protocols are needed to confirm the findings and assess their clinical utility. Due to the small number of studies, their heterogeneity, and the lack of long-term results, trends in the observed changes cannot be clearly determined.

When treating Class II patients with aligners, regular TMJ assessment is recommended, especially in patients who experienced any symptoms (crackling, crepitus, pain) prior to treatment. In the case of minor malocclusions, CAs may be the preferred tool for patients without TMJ dysfunction (especially when precise movement control methods are introduced to limit undesirable co-movements) due to the predictability and control of orthodontic forces, which reduces the risk of sudden occlusal changes and promotes neuromuscular adaptation. In patients with joint complaints, caution and close monitoring are necessary, even after treatment, due to the lack of long-term results. If symptoms occur, they should be promptly recognized and diagnosed using available imaging methods, preferably those that are radiation-free, such as MRI. Further research should focus on long-term patient follow-up, the use of soft tissue imaging, and a clear definition of the impact of aligner treatment on joint structure and function.

## Figures and Tables

**Table 1:** Eligibility criteria.

	Inclusion Criteria	Exclusion Criteria
Patient	Patient under orthodontic treatment	
Intervention	Distalization using clear aligners	Different types of appliances
Control	None	None
Outcome	CBCT measurement of changes	Lack of CT or MRI
Timeframe	No limit	Not applicable
Study design	Retrospective cohort study or case report or case series	Non-original/Non-English research, reviews, book chapters, or book fragments

**Table 2:** Joint parameters and methods of their measurement.

Data Item	Description
MFH	The perpendicular line between the most superior central point of the fossa and the lowest point of the articular eminence and the posterior margin of the mandibular fossa.
MFW	The shortest distance between the most anteroinferior point of the mandibular fossa and the most posteroinferior point of the mandibular fossa.
CL	Distance between the most lateral and medial points of the condylar head.
CW	Distance between the most anterior and posterior points of the condylar head.
AJS	The shortest distance between the most anterior condyle point near the most Anterior Joint Space and the most posterior point of the anterior wall of the glenoid fossa.
PJS	The shortest distance between the most posterior condyle point near the Posterior Joint Space and the most anterior point of the posterior wall of fossa.
SJS	The shortest distance between the most superior central point of the condylar head and the most superior central point of the fossa.
MJS	The shortest distance between the most lateral point of the fossa and the innermost point of the condylar head.

**Table 3:** Risk of bias in studies.

Author Year	Temporal Precedence	Selection and Allocation	Confounding Factors	Administration of Intervention/Exposure	Assessment, Detection, and Measurement of the Outcome			Participant Retention	Statistical Conclusion Validity	Overall
Al-Somairi, 2025 [23]	Yes	Yes	Yes	Yes	Yes	Yes	Yes	Yes	Yes	Low risk
Al-Tayar, 2023 [36]	Yes	No	Yes	Yes	Yes	Yes	Yes	Yes	Yes	Moderate risk
Al-Worafi, 2024 [5]	Yes	Yes	Yes	Yes	Yes	Yes	Yes	Yes	Yes	Low risk
Zheng, 2023 [22]	Yes	Yes	Yes	Yes	Yes	Yes	Yes	Yes	Yes	Low risk

**Table 4:** Study characteristics.

Author	Number of Patients (M/F)	Patient’s Age	Imaging Type	Malocclusion Before Treatment	Time of Treatment	
Al-Somairi, 2025 [23]	30 (13/17)	24.27	CBCT	Class I	Mean	2.21
					SD	0.74
Al-Tayar, 2023 [36]	23 (7/16)	29.8	CBCT	Class I or II	Mean	1.97
					SD	0.6
Al-Worafi, 2024 [5]	35 (N/S)	N/S	CBCT	Class II	Mean	2.04
					SD	0.59
Zheng, 2023 [22]	22 (10/12)	23.18	CBCT	Class II division 2	Mean	1.9
					SD	0.18

N/S—Not Specified; CBCT—Cone Beam Computed Tomography; SD—standard deviation.

**Table 5:** Dimensions of selected joint parameters (MFH, MFW, CL, and CW) in included studies.

Author		Mandibular Fossa Dimension MFH (mm)		Mandibular Fossa Dimension MFW (mm)		Condylar Dimension CL (mm)		Condylar Dimension CW (mm)	
		Before	After	Before	After	Before	After	Before	After
Al-Somairi, 2025 [23]	Mean	11.67	11.56	17.47	17.51	19.09	19.15	8.05	8.17
	SD	1.44	2.64	1.67	1.63	1.52	1.47	0.88	1.19
Al-Tayar, 2023 [36]	Mean	8.19	7.75	16.17	18.78	18.94	19.06	10.01	9.27
	SD	1.07	1.08	1.42	1.93	1.16	1.06	0.79	0.18
Al-Worafi, 2024 [5]	Mean	9.49	9.48	15.68	15.69	18.23	18.32	12.44	12.81
	SD	1.37	1.31	1.14	1.41	2.34	2.4	2.1	2.29
Zheng, 2023 [22]	Mean	11.36	11.7	24.83	24.85	16.78	17.26	7.03	7.44
	SD	0.8	0.7	2.09	2.01	1.4	1.41	0.87	0.91

SD—standard deviation; MFH—Mandibular Fossa Height; MFW—Mandibular Fossa Width; CL—Condylar Length; CW—Condylar Width.

**Table 6:** Dimensions of selected joint parameters (AJS, SJS, PJS, and MJS) in included studies.

Author		TMJ Spaces AJS (mm)		TMJ Spaces SJS (mm)		TMJ Spaces PJS (mm)		TMJ Spaces MJS (mm)	
		Before	After	Before	After	Before	After	Before	After
Al-Somairi, 2025 [23]	Mean	2.55	2.6	3.91	3.77	2.88	2.77	3.29	3.34
	SD	0.69	0.76	0.94	0.97	0.56	0.46	0.83	0.92
Al-Tayar, 2023 [36]	Mean	2.68	2.78	2.63	3.19	2.73	1.79	4.67	4.36
	SD	1.16	1.06	0.22	0.02	1.31	0.19	0.92	0.66
Al-Worafi, 2024 [5]	Mean	2.62	2.55	3.97	3.94	2.7	2.73	4.5	4.49
	SD	0.69	0.66	1.05	0.93	0.71	0.76	1.5	1.46
Zheng, 2023 [22]	Mean	2.55	2.33	2.85	3.55	1.99	2.35	2.85	2.86
	SD	0.35	0.33	0.44	0.57	0.37	0.38	0.5	0.46

SD—standard deviation; AJS—Anterior Joint Space; SJS—Superior Joint Space; PJS—Posterior Joint Space; MJS—Medial Joint Space.

**Table 7:** Comparison of mean differences for TMJ measurements reported by individual authors.

Dimension	Author	Number of Patients	Mean Difference	Standard Error	Lower 95% Confidence Interval	Upper 95% Confidence Interval	Significance Level
TMJ spaces PJS (mm)	Al-Somairi, 2025 [23]	30	−0.110	0.132	−0.3749	0.1549	0.4092
	Al-Tayar, 2023 [36]	23	−0.940	0.276	−1.4963	−0.3837	0.0014 *
	Al-Worafi, 2024 [5]	35	0.030	0.176	−0.3208	0.3808	0.8650
	Zheng, 2023 [22]	22	0.360	0.113	0.1318	0.5882	0.0027 *
TMJ spaces MJS (mm)	Al-Somairi, 2025 [23]	30	0.050	0.226	−0.4028	0.5028	0.8259
	Al-Tayar, 2023 [36]	23	−0.310	0.236	−0.7858	0.1658	0.1960
	Al-Worafi, 2024 [5]	35	−0.010	0.354	−0.7160	0.6960	0.9775
	Zheng, 2023 [22]	22	0.010	0.145	−0.2823	0.3023	0.9453
TMJ spaces SJS (mm)	Al-Somairi, 2025 [23]	30	−0.140	0.247	−0.6336	0.3536	0.5724
	Al-Tayar, 2023 [36]	23	0.560	0.046	0.4672	0.6528	<0.0001 *
	Al-Worafi, 2024 [5]	35	−0.030	0.237	−0.5031	0.4431	0.8997
	Zheng, 2023 [22]	22	0.700	0.154	0.3902	1.0098	<0.0001 *
TMJ spaces AJS (mm)	Al-Somairi, 2025 [23]	30	0.050	0.187	−0.3251	0.4251	0.7906
	Al-Tayar, 2023 [36]	23	0.100	0.328	−0.5603	0.7603	0.7617
	Al-Worafi, 2024 [5]	35	−0.070	0.161	−0.3921	0.2521	0.6659
	Zheng, 2023 [22]	22	−0.220	0.103	−0.4270	−0.0130	0.0378 *
Mandibular fossa dimension MFH (mm)	Al-Somairi, 2025 [23]	30	−0.11	0.549	−1.2090	0.9890	0.8419
	Al-Tayar, 2023 [36]	23	−0.440	0.317	−1.0789	0.1989	0.1721
	Al-Worafi, 2024 [5]	35	−0.010	0.320	−0.6494	0.6294	0.9752
	Zheng, 2023 [22]	22	0.340	0.222	−0.1067	0.7867	0.1322
Mandibular fossa dimension MFW (mm)	Al-Somairi, 2025 [23]	30	0.040	0.426	−0.8129	0.8929	0.9255
	Al-Tayar, 2023 [36]	23	2.610	0.500	1.6031	3.6169	<0.0001 *
	Al-Worafi, 2024 [5]	35	0.010	0.306	−0.6016	0.6216	0.9741
	Zheng, 2023 [22]	22	0.020	0.618	−1.2276	1.2676	0.9743
Condylar dimension CL (mm)	Al-Somairi, 2025 [23]	30	0.060	0.386	−0.7128	0.8328	0.8770
	Al-Tayar, 2023 [36]	23	0.120	0.328	−0.5403	0.7803	0.7159
	Al-Worafi, 2024 [5]	35	0.090	0.567	−1.0406	1.2206	0.8743
	Zheng, 2023 [22]	22	0.480	0.424	−0.3749	1.3349	0.2636
Condylar dimension CW (mm)	Al-Somairi, 2025 [23]	30	0.120	0.270	−0.4209	0.6609	0.6586
	Al-Tayar, 2023 [36]	23	−0.740	0.169	−1.0805	−0.3995	0.0001 *
	Al-Worafi, 2024 [5]	35	0.370	0.525	−0.6780	1.4180	0.4835
	Zheng, 2023 [22]	22	0.410	0.268	−0.1317	0.9517	0.1341

*—statistically significant.

**Table 8:** Matrix correlation Spearman test.

Variable	PJS	MJS	SJS	AJS	MFH	MFW	CL	CW
PJS	1	0.4	0.4	−1	1	−0.8	0.4	1
MJS	0.4	1	−0.4	−0.4	0.4	−0.2	−0.4	0.4
SJS	0.4	−0.4	1	−0.4	0.4	0	1	0.4
AJS	−1	−0.4	−0.4	1	−1	0.8	−0.4	−1
MFH	−1	0.4	0.4	−1	1	−0.8	0.4	1
MFW	−0.8	−0.2	0	0.8	−0.8	1	0	−0.8
CL	0.4	−0.4	1	−0.4	0.4	0	1	0.4
CW	1	0.4	0.4	−1	1	−0.8	0.4	1

AJS—Anterior Joint Space; CL—Condylar Length; CW—Condylar Width; MFH—Mandibular Fossa Height; MFW—Mandibular Fossa Width; MJS—Medial Joint Space; PJS—Posterior Joint Space; SJS—Superior Joint Space.

## Data Availability

All data are contained within this article.

## Abbreviations

The following abbreviations are used in this manuscript:

AJS	Anterior Joint Space
CAs	Clear Aligners
CBCT	Cone Beam Computed Tomography
CL	Condylar Length
CW	Condylar Width
FAs	Fixed Appliances
MFH	Mandibular Fossa Height
MFW	Mandibular Fossa Width
MJS	Medial Joint Space
N/A	Not Applicable
PJS	Posterior Joint Space
SD	Standard Deviation
SJS	Superior Joint Space
TMDs	Temporomandibular Disorders
TMJ	Temporomandibular Joint

## Appendix A

**Table A1:** Search engine scopes.

Engine	Scope, records
Embase	over 45,600,000
Web of Science	over 235 million
BASE	418.356.513
PubMed	over 38 million
Scopus	over 100 million

**Table A2:** Reports excluded at the full-text assessment stage.

First Author, Publication Year	Identifier	Reason for Exclusion
[65]	https://pubmed.ncbi.nlm.nih.gov/36643222/ (accessed on 16 April 2025)	Inhomogeneous data
[66]	https://www.sciencedirect.com/science/article/pii/S2666430524001055 (accessed on 16 April 2025)	Inhomogeneous data
[67]	https://bmcoralhealth.biomedcentral.com/articles/10.1186/s12903-022-02526-2 (accessed on 18 April 2025)	Outcome
[68]	https://repository.ucc.edu.co/entities/publication/64c941ba-d029-4157-a214-0ebf71612e20 (accessed on 18 April 2025)	Patient
